# Enterovirus A71 Vaccines

**DOI:** 10.3390/vaccines9030199

**Published:** 2021-02-27

**Authors:** Mei-Ling Li, Shin-Ru Shih, Blanton S. Tolbert, Gary Brewer

**Affiliations:** 1 Department of Biochemistry and Molecular Biology, Rutgers Robert Wood Johnson Medical School, Piscataway, NJ 08854, USA; brewerga@rwjms.rutgers.edu; 2 Research Center for Emerging Viral Infections, College of Medicine, Chang Gung University, Taoyuan 333, Taiwan; srshih@mail.cgu.edu.tw; 3 Department of Laboratory Medicine, Linkou Chang Gung Memorial Hospital, Taoyuan 333, Taiwan; 4 Department of Medical Biotechnology and Laboratory Science, College of Medicine, Chang Gung University, Taoyuan 333, Taiwan; 5 Research Center for Chinese Herbal Medicine, Research Center for Food and Cosmetic Safety, and Graduate Institute of Health Industry Technology, College of Human Ecology, Chang Gung University of Science and Technology, Taoyuan 333, Taiwan; 6 Department of Chemistry, Case Western Reserve University, Cleveland, OH 44106, USA; bst18@case.edu

**Keywords:** Enterovirus A71, hand, foot and mouth disease (HFMD), vaccine, Sinovac

## Abstract

Enterovirus A71 (EV-A71) is a major causative agent of hand, foot, and mouth disease (HFMD) and herpangina. Moreover, EV-A71 infection can lead to neurological complications and death. Vaccination is the most efficient way to control virus infection. There are currently three inactivated, whole EV-A71 vaccines licensed by the China NMPA (National Medical Products Administration). Several other types of vaccines, such as virus-like particles and recombinant VP1 (capsid protein), are also under development. In this review, we discuss recent advances in the development of EV-A71 vaccines.

## 1. Introduction

Enterovirus A71 (EV-A71) is one of the most important neurotropic viruses and poses a serious threat to public health worldwide. EV-A71 is a major causative agent of hand, foot, and mouth disease (HFMD) which is highly contagious. EV-A71 infection in young children can also lead to neurological complications, rapid fatal pulmonary edema, myocarditis, hemorrhage, and death [1,2,3,4,5]. The absence of broadly protective vaccines and effective antivirals makes EV-A71 an important pathogen of public health concern. EV-A71 is a member of the genus Enterovirus within the family *Picornaviridae*. EV-A71 consists of three distinct genotypes (A, B, and C). Genotype B is divided into subgenotypes B1 to B5. Genotype C is divided into subgenotypes C1 to C5, and C4 is further divided into C4 a and C4 b. Subgenotypes B3, B4, C1, and C2 co-circulated in the Asia–Pacific region from 1999 to 2016. C4 is the most prevalent genotype circulating in China; C1 and C2 are most prevalent in Europe while B4 and B5 are most prevalent in other regions [6,7,8].

EV-A71 is a non-enveloped virus containing a positive-stranded RNA genome ~7400 nucleotides in length. The viral genome within its icosahedral capsid is flanked by highly structured 5′ and 3′ untranslated regions (UTR) and is polyadenylated at its 3′ end. The large single polyprotein encoded by the viral genome is subsequently processed by viral proteases 2 A and 3 C into P1, P2, and P3.

**Figure 1:**
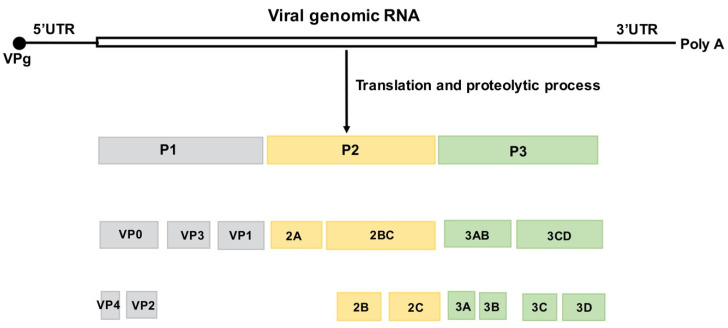
Structure of the EV-A71 genome and the encoded viral proteins. The single open reading frame (ORF) is flanked by a highly structured 5′UTR and 3′UTR followed by a poly (A) tail. The 5′ end of the viral genome is covalently bound to the viral VPg protein. The ORF is divided into three regions. P1 encodes four structural (capsid) proteins, VP1–4. P2 and P3 encode seven nonstructural proteins, 2 A to 2 C and 3 A to 3 D, respectively [11].

P1 is further processed to produce four capsid proteins—VP1 to 4. P2 and P3 are processed into seven nonstructural proteins—2 A−2 C and 3 A−3 D—for viral replication (Figure 1). The VP1 protein contains the major neutralization epitopes that are used in viral serotype identification and phylogenic studies [9,10].

Currently, there is no US FDA-approved antiviral agent or vaccine against EV-A71. However, there are three inactivated, EV-A71 whole-virus vaccines approved by the China National Medical Products Administration (NMPA) and are commercially available in China. Large-scale clinical trials showed that vaccine efficacy against EV-A71-associated Hand, foot, and mouth disease (HFMD) was at least 90%. Vaccine effectiveness in HFMD patients under five years old was 100% for severe cases and ~80% for mild cases, suggesting that the vaccine performs well in practice [8,12]. Vaccination is the most effective countermeasure against EV-A71 infection and epidemic. In this review, we summarize the recent progress in the development of vaccines against EV-A71 and discuss the prospects and challenges in this field.

## 2. Whole EV-A71 Vaccines

### 2.1. Inactivated Whole EV-A71 Vaccines

Inactivation of whole virus is the conventional and safest way to produce viral vaccine [13]. There are three inactivated whole virus EV-A71 vaccines that completed clinical trials and were licensed in China in 2015 [14,15]. However, the US FDA did not approve the vaccines due to concerns about the effectiveness against different pandemic strains, safety, and quality control of vaccine production. The three formalin-inactivated EV-A71 vaccines were developed by three different institutions: Sinovac Biotech, Beijing Vigoo, and Chinese Academy of Medical Science (CAMS). The three companies established their own seed lot system and cell banks. CAMS used diploid cells KMB−17 derived from human fetal lung, as a cell bank and the cells were grown in a cell factory, whereas Vigoo and Sinovac used Vero cells in a microcarrier bioreactor and cell factory, respectively. The screening and evaluation of virus strains used for vaccine production were carefully performed to select strains that could induce cross-protecting antibodies. Two animal models have been developed to study EV-A71 vaccines. One is the suckling monkey (rhesus) model which is used to study EV-A71 infection and pathogenicity, and the other is the suckling mouse model which is used to evaluate vaccine immunogenicity. The protective efficacy of EV-A71 vaccines was demonstrated using both animal models. The results of the animal model studies provided the rationale for proceeding with clinical trials [16,17]. The EV-A71 C4 subgenotype was chosen as the virus seed because it is the most prevalent genotype circulating in China [10,18].

The safety and efficacy of inactivated EV-A71 vaccines had been confirmed in large phase III clinical trials (Table 1). Vaccine efficacy was at least 90% against EV-A71-associated HFMD and 100% against EV-A71-associated HFMD with neurologic complications [19].

CAMS, Vigoo, and Sinovac (Beijing, China) initiated the phase I trials between December 2010 and Feburary 2011. The phase III trials were completed in 2013. CAMS recruited 12,000 infants and children of 6 to 71 months of age for phase III clinical trials (ClinicalTrials.gov number: NCT01569581). Children randomly received two doses of 100 U (EV-A71 antigen unit) CAMS vaccine or placebo four weeks apart and were followed for two years. The EV-A71 antigen contents were measured by a quantitative ELISA. Based on the EV-A71 antigen reference standard (1600 U/mL) established by the China NMPA, the specific activity of EV-A71 antigen is 421.1 U/μg [20]. Vaccine efficacy against EV-A71-associated HFMD was 97.4% [21]. The Vigoo phase III clinical trial (ClinicalTrials.gov number: NCT01508247) has recruited 10,245 infants and children between 6 and 35 months of age. The participants were randomly given 320 U vaccine or placebo on days 1 and 28. The participants were followed for two years [22,23].The efficacy of the vaccine against EV-A71 induced HFMD was 90% at the first year and reached 100 % at the second year. The efficacy of the vaccine against other illness associated with EV-A71 infection was 80.4% at the first year. The vaccine did not cause any serious adverse effects.

In the Sinovac (Beijing, China) phase III trial (NCT01507857), 10,077 children aged 6 to 35 months were given 400 U of Sinovac vaccine or placebo at days 1 and 28 and were followed for two years. At the end of the first year, the vaccine efficacy against EV-A71-induced HFMD and herpangina was 94.8%. The efficacy against EV-A71-associated HFMD that manifested to neurological complications was 100%. At the end of the second year, the vaccine efficacy against EV-A71-associated HFMD was 95.1% [19]. Sinovac then conducted a five-year follow-up survey to study the long-term immunogenicity. The study results showed that the Sinovac vaccine demonstrated the long-term persistence of immunogenicity within five years [24]. During this time, the Sinovac EV-A71 vaccine production capacity had reached more than 20 million doses/year. WHO organized a working group meeting in 2019 to develop WHO recommendations to assure the efficacy, safety and quality of EV-A71 vaccines. In view of its cross-reactivity with different genotypes of EV-A71, WHO recommendations suggested that the EV-A71-C4 a vaccine could be used worldwide [25].

The National Health Research Institutes (NHRI) (Hsinchu, Taiwan)in Taiwan used a subgenotype B4 strain to produce the inactivated whole virus EV-A71 vaccine [26,27]. For the vaccine safety and immunogenicity study in the phase I clinical trial (ClinicalTrials.gov number: NCT01268787) sixty healthy participants aged 20 to 60 years were recruited and randomly given 5 μg or 10 μg of EV-A71 antigen on days 1 and 21. The study showed that the NHRI EV-A71 vaccine elicited antibodies against EV-A71 without adverse effects [28]. The antibodies produced in most of the participants showed cross-neutralization against subgenotypes B1, B5, and C4 [29,30]. In phase II trials (ClinicalTrials.gov numbers: NCT02777411, NCT03268083 and NCT02200237) Enimmune Corp. and Medigen Vaccinology Corp. in Taiwan have continued the safety and immunogenicity evaluation of the inactivated EV-A71 vaccine with low, medium, and high doses. Medigen recruited 365 participants from 2 months to 11 years old and participants were randomly given different doses of vaccine or placebo. The EV-A71 vaccine elicited an immune response against subgenotypes B4, B5, C4 a, C4 b, and C5 without vaccine-related serious adverse events. Vaccine immunity persisted for two years [31]. Based upon the promising results from phase II trials, the companies initiated phase III trials (ClinicalTrials.gov number: NCT03865238) in 2019 which should be completed in 2022.

The Korea National Research Institute of Health (Chungcheongbuk-do, Korea) developed an inactivated whole virus vaccine against EV-A71 using an EV-A71 strain (genotype C4 a) isolated from a Korean patient. [32]. The vaccine was able to induce a strong immunogenic response in mice. The vaccine is currently being evaluated in a blinded, randomized, placebo-controlled phase I study in healthy humans in Korea.

In Thailand, there was an early development of an inactivated EV-A71 vaccine. A genotype C4 EV71-A71 strain was grown in Vero cells in roller bottles. The purified, inactivated virus was able to induce neutralizing antibodies against EV-A71 in BALB/C mice. This prototype product will be further developed into an EV-A71 vaccine candidate [25].

### 2.2. Live-Attenuated Vaccines

Live-attenuated vaccines have the advantages of long-lasting immunity and cost-effective production; however, the molecular pathogenic mechanism of EV-A71 is not fully understood. The EV-A71 virulence determinants have not been completely elucidated. However, several EV-A71 virulence determinants have been identified in the 5′UTR, VP1, VP2, 2 A, 3 C, and 3 D [33]. One of the key virulence determinants is the amino acid at position 145 of VP1. Changing glutamine to glutamic acid at this position increased the virulence in monkeys [34,35]. The highly structured 5′UTR plays an important role in viral translation and virulence. Changing cytosine to uridine at position 158 in stem-loop II of the 5′UTR reduced EV-A71 viral translation and virulence in a mouse model [36]. Chang et al. [37] grew EV-A71 in Vero cells and then passaged it in RD cells to select mutant virus adapted to RD cells. There were three amino acid changes, E145 G, V146 I, and S241 L, in VP1 and a single nucleotide mutation at nt 494 of the 5′UTR of the selected mutant virus. These mutations contributed to the virulence attenuation of the mutant virus in mice.

The vaccine candidate EV7-A71 (S1–3′), a temperature-sensitive mutant of the prototype BrCr, demonstrated induction of an efficient immune response to a lethal dose of EV-A71 (BrCr-TR) (a temperature-resistant mutant of BrCr) in cynomolgus monkeys. However, inoculation of EV-A71 (S1–3′) caused neurological symptoms in monkeys. Four and 10 days after inoculation, infectious virus was isolated from the monkeys’ lumbar spinal cords. This raised serious safety concerns about this vaccine candidate [38].

Increasing the fidelity of the 3 D RNA-dependent RNA polymerase enhances the safety and stability of live-attenuated vaccines. G64 R, G64 T, and S264 L mutations in 3 D enhanced the polymerase fidelity. G64 R and L123 F mutations in 3 D polymerase attenuated EV-A71 virulence in mice [39,40]. Based upon the study, Tsai et al. constructed a mutant virus with codon deoptimized VP1 and with G64 R and L123 F mutations in the 3 D polymerase to increase the fidelity. This virus was less virulent in a mouse model [41]. Yee et al. [42] constructed a miRNA-based EV-A71 vaccine strain (pIY) carrying let−7 a and miR−124 a target genes in the EV-A71 genome, which has an 11-nucleotide deletion in the 5′UTR and a G64 R mutation in the 3 D RNA-dependent RNA polymerase. The pIY strain’s viral RNA copy number and viral titers were much lower than that of the EV-A71 wild-type B4 strain in SHSY−5 Y cells, which express both let−7 a and miR−124 a. The pIY strain protected mice against a lethal dose of EV-A71.

## 3. VLP (Virus Like Particle)-Based Vaccines

VLP (virus-like particle) is a promising vaccine candidate because it resembles the authentic virus in appearance and antigenicity. VLP can elicit innate and adaptive immunity and is safer because the lack of a viral genome makes them incapable of replicating in the host. Several VLP-based vaccines, such as Recombivax HB and Engerix-B for hepatitis B virus, and Gardasil, Cervarix, and Gardasil−9 for human papillomavirus, have been licensed [43,44].

Several strategies have been employed to produce EV-A71 VLPs. The baculovirus expression system is widely used for the production of VLPs. To produce EV-A71 VLPs, insect cells were infected with a recombinant baculovirus co-expressing the EV-A71 P1 region and the viral protease 3 CD from various promoters [45,46]. The P1 region is cleaved by the 3 CD protease to produce the viral capsids VP1–4. Baculovirus-produced EV-A71 VLPs protected monkeys from EV-A71 infection, suggesting that the VLP is a promising vaccine candidate [47]. However, the yield of EV-A71 VPLs was low due to their degradation. To increase the yield and purity, Zhao et al. [43] purified the baculovirus-expressed EV-A71 VLPs using Capto Core 700, Capto Adhere resin, and Capto Butyl columns (GE Healthcare). The multistep chromatography process resulted in EV-A71 VLPs with ~31.52% yield and >95% purity. An occupied N-glycosylation site was detected in VP1 using MALDI-TOF/TOF. Glycosylation is the most important post-translational modification of proteins and plays a critical role in antigen immunogenicity [48]. Hepatitis B virus (HBV) VLPs with additional N-glycosylation sites, induced enhanced immunogenicity [49]. Therefore, further study is needed to verify whether the identified N-glycosylation site on VP1 residue N176 is essential for an immune response. Nevertheless, EV-A71 VLPs could induce a high titer of neutralizing antibodies that protected newborn mice from a lethal challenge of the EV-A71 C4 strain.

Zhang et al. [50] improved the yield by expressing EV-A71 VLPs in the yeast *Pichia pastoris*. The EV-A71 VLP yield was high and potently induced neutralizing antibodies against various strains of EV-A71 in a mouse model [51]. More importantly, maternal immunization with VLPs protected neonatal mice against a lethal EV-A71 challenge [50,51].

Tsou et al. and Yan et al. used Adenovirus and Vesicular stomatitis virus-based vaccines on expressed EV-A71 VLPs in mammalian cells. Both VLPs elicited antibodies against EV-A71 infection in mice [52,53].

Hand, foot, and mouth disease (HFMD) is highly contagious and has led to significant morbidity and mortality worldwide [54]. EV-A71, CVA6 (coxsackievirus A6), CVA10, and CVA16 are major causative agents of HFMD. However, the inactivated EV-A71 whole virus vaccine failed to cross-protect against infections by other HFMD-causing enteroviruses. To develop a multivalent HFMD vaccine, Zhang et al. [55] used a baculovirus expression system to generate recombinant VLPs of EV-A71, CVA6, CVA10, and CVA16 and then combined EV-A71-VLP, CVA6-VLP, CVA10-VLP, and CVA16-VLP to formulate a tetravalent VLP vaccine. The antisera resulting from immunization with the tetravalent VLP vaccine protected mice from single or co-infection of EV-A71, CVA6, CVA10, and CVA16, suggesting that it is a promising HFMD vaccine candidate.

## 4. Recombinant VP1 and P1 Vaccines

The EV-A71 VP1 capsid protein contains neutralization epitopes. Several expression strategies, such as *Escherichia coli* (*E. coli*), baculovirus, and HIV gag-based VLP carrier were used to express recombinant VP1 protein. The purified recombinant VP1 protein protected mice from EV-A71 infection; however, the protection was lower than that of inactivated EV-A71 vaccine [56,57,58,59].

Han et al. [60] expressed the modified EV-A71 polyprotein P1 in *Pichia pastoris* as a vaccine candidate. P1 protein induced persistent, high cross-neutralization antibodies for different EV-A71 subtypes in rabbits. Vaccination of pregnant mice cross-protected neonatal mice against different subtypes of EV-A71. EV-A71 antibody production elicited by P1 was one week later than that of inactivated EV-A71 virus. However, the level and duration of EV-A71 antibody production was stronger than that of inactivated EV-A71 virus. This could be related to antigen glycosylation [48] as studies showed that modification of the glycosylation sites of HCV E1 protein and HIV gp120 enhanced the immune response [61,62]. With strong immunogenicity and cross-protection against different EV-A71 subtypes, the *P. pastoris*-expressed P1 protein is a promising EV-A71 vaccine candidate.

## 5. Synthetic Peptide Vaccines

Synthetic peptides are safe and efficacious for multivalent vaccine development. Several groups have focused on mapping the antigen epitopes in EV-A71 capsid proteins (VP1–4).

Foo et al. [63] used synthetic, overlapping peptides spanning VP1 to map antigen epitopes. The study identified two epitopes—SP55 and SP70 (amino acids 163–177 and 208–222 in VP1, respectively). Both peptides elicited antibodies that protected mice from EV-A71 infection. Anti-SP70 antisera passively protected suckling mice against various EV-A71 strains [64].

Liu et al. [65] selected six peptides (P70–159 in VP2, P140–249 in VP2, P324–443 in VP3, P444–565 in VP3, P566–665 in VP1, and P746–876 in VP1) that individually protected from EV-A71 infection. They combined these into three vaccine candidates for further evaluation in neonatal mice. The studies showed that a combination of four synthetic peptides (P70–159 in VP2, P140–249 in VP2, P324–443 in VP3, and P746–876 in VP1) of the structural proteins provided effective protection of newborn mice against EV-A71 infection.

Aw-Yong et al. [66] synthesized 63 peptides spanning the four structural and seven non-structural proteins of EV-A71 to map the potential epitopes. The study showed that amino acids 41–55 in VP1 was an EV-A71 IgG-specific epitopeamino acids 142–156 in VP1 was recognized as the EV-A71 IgM-specific immunodominant epitope.

Several groups used a fusion protein strategy to express EV-A71 epitopes. Xu et al. [67] fused EV-A71 VP2 (amino acids 141–155) epitope with hepatitis B virus core protein to generate HBc-VP2 (amino acids 141–155). This fusion protein induced cross-neutralizing antibodies against EV-A71 and protected newborn mice from EV-A71 infection. Huo et al. [68] fused EV-A71 VP1 epitope (amino acids 208–222) and CV-A16 VP1 epitope (amino acids 271–285) with hepatitis B virus core protein. The expressed epitopes induced an immune response and protected suckling mice against EV-A71 and CV-A16 infection. Jiang et al. [69] fused EV-A71 VP3 epitope (amino acids 176–190) with the P domain of norovirus capsid protein. The fusion protein elicited an immune response and protected suckling mice from a lethal dose of EV-A71 infection. Recently, Mustafa et al. [70] fused truncated EV-A71 VP1 (amino acids 198–297) with Newcastle disease virus capsid protein and expressed it in *E. coli*. The recombinant protein elicited neutralizing antibodies against EV-A71 in a mouse model.

## 6. Conclusions and Prospective

EV-A71 is a major causative agent of HFMD, and EV-A71 infection has also led to neurological complications and death in young children worldwide. Vaccines are the most effective way to prevent EV-A71 infection.

The primary strategy to develop EV-A71 vaccines is to use viral structural proteins as immunogens. The inactivated whole-virus vaccine, live-attenuated virus vaccine, VLP vaccine, recombinant VP1 vaccine, and synthetic peptide vaccine all deliver wholly or partially expressed viral proteins to the host to elicit host immunogenicity and produce neutralizing antibodies. The inactivated, whole-virus vaccines yield high immunogenicity levels with high neutralization titers and induce cross-genotype neutralizing antibody responses more effectively. Currently, there are three inactivated, whole-virus vaccines against EV-A71 approved by the China NMPA, and WHO recommendations believe the Sinovac vaccine could be used worldwide [25]. However, the inactivated EV-A71 vaccine still faces two major challenges-cross-genotype and long-term protection. Although the Vigoo, Sinovac, and CAMS vaccines (using the C4 genotype) and the NHRI vaccine (using the B4 genotype) showed cross-protection against current circulating EV-A71 strains, the B4-based vaccine poorly neutralized a C2 isolate. Although the study by Hu et al. observed that the neutralizing antibodies elicited by inactivated EV-A71 vaccine persisted for five years, immunity decreased after six months [8,24].

The advantages of recombinant VP1 and synthetic peptide vaccines are that they are safer and more cost-effective. These vaccines reduce the risk of unwanted immune responses such as antibody-dependent enhancement (ADE). ADE is a general concern for vaccine design because the mechanisms underlying antibody protection against any pathogen infection have a theoretical potential to amplify the infection or trigger harmful immune responses [71]. However, the recombinant VP1, synthetic peptide, and VLP vaccines elicit low levels of virus-neutralizing antibody responses and require strong adjuvants [29]. If these vaccine candidates can be refined to boost immunogenicity levels, they could become powerful therapeutic options. For example, with the availability of the X-ray crystallographic structure of VP1 [72], bioinformatics, proteomics, and immunology can be applied to develop peptide-based, synthetic vaccines. These techniques can facilitate the design of potent peptides that contain virus-neutralization epitopes and efficiently induce high levels of virus-neutralizing antibody responses.

mRNA vaccines represent a promising alternative vaccine platform technology because of their high potency, rapid development, completely synthetic nature, low-cost manufacturing process, and safe administration. Nine months after the COVID−19 pandemic began, the US government granted emergency-use authorization to two COVID−19 mRNA vaccines—Pfizer-BioNTech [73] and Moderna mRNA−1273 [74,75]. It is also worth the effort to develop mRNA vaccines against EV-A71 in the future.

## Figures and Tables

**Table 1:** China-licensed inactivated whole EV-A71 vaccines.

Org.	Cells	Dosage	Phase III	Age	Efficacy	Approval Date
**CAMS**	KMB-17	100 U	March 2012–Februaury 2013	6–71 mo	97.4%	December 2015
**Vigoo**	Vero	320 U	January 2012–March 2013	6–35 mo	90%	December 2016
**Sinovac**	Vero	400 U	January 2012–March 2013	6–35 mo	94.8%	December 2015

## References

[1] Alexander J.P., Baden L., Pallansch M.A., Anderson L.J. (1994). Enterovirus 71 infections and neurologic disease--United States, 1977–1991. J. Infect. Dis..

[2] Ooi M.H., Wong S.C., Lewthwaite P., Cardosa M.J., Solomon T. (2010). Clinical features, diagnosis, and management of enterovirus 71. Lancet Neurol..

[3] Lee K.Y. (2016). Enterovirus 71 infection and neurological complications. Korean J. Pediat..

[4] Chang L.Y., Lin H.Y., Gau S.S., Lu C.Y., Hsia S.H., Huang Y.C., Huang L.M., Lin T.Y. (2019). Enterovirus A71 neurologic complications and long-term sequelae. J. Biomed. Sci..

[5] Puenpa J., Wanlapakorn N., Vongpunsawad S., Poovorawan Y. (2019). The History of Enterovirus A71 Outbreaks and Molecular Epidemiology in the Asia-Pacific Region. J. Biomed. Sci..

[6] Nikonov O.S., Chernykh E.S., Garber M.B., Nikonova E.Y. (2017). Enteroviruses: Classification, Diseases They Cause, and Approaches to Development of Antiviral Drugs. Biochemistry..

[7] Kung Y.A., Hung C.T., Liu Y.C., Shih S.R. (2014). Update on the development of enterovirus 71 vaccines. Expert Opin. Biol. Ther..

[8] Chang Y.K., Chen K.H., Chen K.T. (2018). Hand, foot and mouth disease and herpangina caused by enterovirus A71 infections: A review of enterovirus A71 molecular epidemiology, pathogenesis, and current vaccine development. Rev. Inst. Med. Trop. Sao Paulo.

[9] Chen B.S., Lee H.C., Lee K.M., Gong Y.N., Shih S.R. (2020). Enterovirus and Encephalitis. Front. Microbiol..

[10] Lin J.Y., Kung Y.A., Shih S.R. (2019). Antivirals and vaccines for Enterovirus A71. J. Biomed. Sci..

[11] Shih S.R., Stollar V., Li M.L. (2011). Host factors in enterovirus 71 replication. J. Virol..

[12] Wang X., An Z., Huo D., Jia L., Li J., Yang Y., Liang Z., Wang Q., Wang H. (2019). Enterovirus A71 vaccine effectiveness in preventing enterovirus A71 infection among medically-attended hand, foot, and mouth disease cases, Beijing, China. Hum. Vaccin. Immunother..

[13] Minor P.D. (2004). Polio eradication, cessation of vaccination and re-emergence of disease. Nat. Rev. Microbiol..

[14] Zhou Y., Li J.X., Jin P.F., Wang Y.X., Zhu F.C. (2016). Enterovirus 71: A whole virion inactivated enterovirus 71 vaccine. Expert Rev. Vaccin..

[15] Mao Q.Y., Wang Y., Bian L., Xu M., Liang Z. (2016). EV71 vaccine, a new tool to control outbreaks of hand, foot and mouth disease (HFMD). Expert Rev. Vaccin..

[16] Bek E.J., Hussain K.M., Phuektes P., Kok C.C., Gao Q., Cai F., Gao Z., McMinn P.C. (2011). Formalin-inactivated vaccine provokes cross-protective immunity in a mouse model of human enterovirus 71 infection. Vaccine.

[17] Dong C., Liu L., Zhao H., Wang J., Liao Y., Zhang X., Na R., Liang Y., Wang L., Li Q. (2011). Immunoprotection elicited by an enterovirus type 71 experimental inactivated vaccine in mice and rhesus monkeys. Vaccine.

[18] Chong P., Liu C.C., Chow Y.H., Chou A.H., Klein M. (2015). Review of enterovirus 71 vaccines. Clin. Infect. Dis. Off. Publ. Infect. Dis. Soc. Am..

[19] Zhu F., Xu W., Xia J., Liang Z., Liu Y., Zhang X., Tan X., Wang L., Mao Q., Wu J. (2014). Efficacy, safety, and immunogenicity of an enterovirus 71 vaccine in China. N. Engl. J. Med..

[20] Liang Z., Mao Q., Gao Q., Li X., Dong C., Yu X., Yao X., Li F., Yin W., Li Q. (2011). Establishing China’s national standards of antigen content and neutralizing antibody responses for evaluation of enterovirus 71 (EV71) vaccines. Vaccine.

[21] Li R., Liu L., Mo Z., Wang X., Xia J., Liang Z., Zhang Y., Li Y., Mao Q., Wang J. (2014). An inactivated enterovirus 71 vaccine in healthy children. N. Engl. J. Med..

[22] Zhu F.C., Meng F.Y., Li J.X., Li X.L., Mao Q.Y., Tao H., Zhang Y.T., Yao X., Chu K., Chen Q.H. (2013). Efficacy, safety, and immunology of an inactivated alum-adjuvant enterovirus 71 vaccine in children in China: A multicentre, randomised, double-blind, placebo-controlled, phase 3 trial. Lancet.

[23] Wei M., Meng F., Wang S., Li J., Zhang Y., Mao Q., Hu Y., Liu P., Shi N., Tao H. (2017). 2-Year Efficacy, Immunogenicity, and Safety of Vigoo Enterovirus 71 Vaccine in Healthy Chinese Children: A Randomized Open-Label Study. J. Infect. Dis..

[24] Hu Y., Zeng G., Chu K., Zhang J., Han W., Zhang Y., Li J., Zhu F. (2018). Five-year immunity persistence following immunization with inactivated enterovirus 71 type (EV71) vaccine in healthy children: A further observation. Hum. Vaccin. Immunother..

[25] Lei D., Griffiths E., Martin J. (2020). WHO working group meeting to develop WHO Recommendations to assure the quality, safety and efficacy of enterovirus 71 vaccines. Vaccine.

[26] Chang J.Y., Chang C.P., Tsai H.H., Lee C.D., Lian W.C., Ih Jen S., Sai I.H., Liu C.C., Chou A.H., Lu Y.J. (2012). Selection and characterization of vaccine strain for Enterovirus 71 vaccine development. Vaccine.

[27] Chong P., Hsieh S.Y., Liu C.C., Chou A.H., Chang J.Y., Wu S.C., Liu S.J., Chow Y.H., Su I.J., Klein M. (2012). Production of EV71 vaccine candidates. Hum. Vaccin. Immunother..

[28] Cheng A., Fung C.P., Liu C.C., Lin Y.T., Tsai H.Y., Chang S.C., Chou A.H., Chang J.Y., Jiang R.H., Hsieh Y.C. (2013). A Phase I, randomized, open-label study to evaluate the safety and immunogenicity of an enterovirus 71 vaccine. Vaccine.

[29] Chou A.H., Liu C.C., Chang C.P., Guo M.S., Hsieh S.Y., Yang W.H., Chao H.J., Wu C.L., Huang J.L., Lee M.S. (2012). Pilot scale production of highly efficacious and stable enterovirus 71 vaccine candidates. PLoS ONE.

[30] Chou A.H., Liu C.C., Chang J.Y., Jiang R., Hsieh Y.C., Tsao A., Wu C.L., Huang J.L., Fung C.P., Hsieh S.M. (2013). Formalin-inactivated EV71 vaccine candidate induced cross-neutralizing antibody against subgenotypes B1, B4, B5 and C4A in adult volunteers. PLoS ONE.

[31] Huang L.M., Chiu C.H., Chiu N.C., Lin C.Y., Li M.T., Kuo T.Y., Weng Y.J., Hsieh E.F., Tai I.C. (2019). Immunogenicity, safety, cross-reaction, and immune persistence of an inactivated enterovirus A71 vaccine in children aged from two months to 11 years in Taiwan. Vaccine.

[32] In H.J., Lim H., Lee J.A., Kim H.J., Kim J.W., Hyeon J.Y., Yeo S.G., Lee J.W., Yoo J.S., Choi Y.K. (2017). An inactivated hand-foot-and-mouth disease vaccine using the enterovirus 71 (C4a) strain isolated from a Korean patient induces a strong immunogenic response in mice. PLoS ONE.

[33] Huang S.W., Cheng D., Wang J.R. (2019). Enterovirus A71: Virulence, antigenicity, and genetic evolution over the years. J. Biomed. Sci..

[34] Fujii K., Sudaka Y., Takashino A., Kobayashi K., Kataoka C., Suzuki T., Iwata-Yoshikawa N., Kotani O., Ami Y., Shimizu H. (2018). VP1 Amino Acid Residue 145 of Enterovirus 71 Is a Key Residue for Its Receptor Attachment and Resistance to Neutralizing Antibody during Cynomolgus Monkey Infection. J. Virol..

[35] Nishimura Y., Lee H., Hafenstein S., Kataoka C., Wakita T., Bergelson J.M., Shimizu H. (2013). Enterovirus 71 binding to PSGL-1 on leukocytes: VP1-145 acts as a molecular switch to control receptor interaction. PLoS Pathog..

[36] Yeh M.T., Wang S.W., Yu C.K., Lin K.H., Lei H.Y., Su I.J., Wang J.R. (2011). A single nucleotide in stem loop II of 5’-untranslated region contributes to virulence of enterovirus 71 in mice. PLoS ONE.

[37] Chang C.K., Wu S.R., Chen Y.C., Lee K.J., Chung N.H., Lu Y.J., Yu S.L., Liu C.C., Chow Y.H. (2018). Mutations in VP1 and 5′-UTR affect enterovirus 71 virulence. Sci. Rep..

[38] Arita M., Nagata N., Iwata N., Ami Y., Suzaki Y., Mizuta K., Iwasaki T., Sata T., Wakita T., Shimizu H. (2007). An attenuated strain of enterovirus 71 belonging to genotype a showed a broad spectrum of antigenicity with attenuated neurovirulence in cynomolgus monkeys. J. Virol..

[39] Sadeghipour S., Bek E.J., McMinn P.C. (2013). Ribavirin-resistant mutants of human enterovirus 71 express a high replication fidelity phenotype during growth in cell culture. J. Virol..

[40] Meng T., Kwang J. (2014). Attenuation of human enterovirus 71 high-replication-fidelity variants in AG129 mice. J. Virol..

[41] Tsai Y.H., Huang S.W., Hsieh W.S., Cheng C.K., Chang C.F., Wang Y.F., Wang J.R. (2019). Enterovirus A71 Containing Codon-Deoptimized VP1 and High-Fidelity Polymerase as Next-Generation Vaccine Candidate. J. Virol..

[42] Yee P.T.I., Tan S.H., Ong K.C., Tan K.O., Wong K.T., Hassan S.S., Poh C.L. (2019). Development of live attenuated Enterovirus 71 vaccine strains that confer protection against lethal challenge in mice. Sci. Rep..

[43] Zhao D., Sun B., Sun S., Fu B., Liu C., Liu D., Chu Y., Ma Y., Bai L., Wu Y. (2017). Characterization of human enterovirus71 virus-like particles used for vaccine antigens. PLoS ONE.

[44] Qian C., Liu X., Xu Q., Wang Z., Chen J., Li T., Zheng Q., Yu H., Gu Y., Li S. (2020). Recent Progress on the Versatility of Virus-Like Particles. Vaccines (Basel).

[45] Chung Y.C., Huang J.H., Lai C.W., Sheng H.C., Shih S.R., Ho M.S., Hu Y.C. (2006). Expression, purification and characterization of enterovirus-71 virus-like particles. World J. Gastroenterol..

[46] Hu Y.C., Hsu J.T., Huang J.H., Ho M.S., Ho Y.C. (2003). Formation of enterovirus-like particle aggregates by recombinant baculoviruses co-expressing P1 and 3CD in insect cells. Biotechnol. Lett..

[47] Lin Y.L., Yu C.I., Hu Y.C., Tsai T.J., Kuo Y.C., Chi W.K., Lin A.N., Chiang B.L. (2012). Enterovirus type 71 neutralizing antibodies in the serum of macaque monkeys immunized with EV71 virus-like particles. Vaccine.

[48] Rudd P.M., Elliott T., Cresswell P., Wilson I.A., Dwek R.A. (2001). Glycosylation and the immune system. Science.

[49] Hyakumura M., Walsh R., Thaysen-Andersen M., Kingston N.J., La M., Lu L., Lovrecz G., Packer N.H., Locarnini S., Netter H.J. (2015). Modification of Asparagine-Linked Glycan Density for the Design of Hepatitis B Virus Virus-Like Particles with Enhanced Immunogenicity. J. Virol..

[50] Zhang C., Ku Z., Liu Q., Wang X., Chen T., Ye X., Li D., Jin X., Huang Z. (2015). High-yield production of recombinant virus-like particles of enterovirus 71 in Pichia pastoris and their protective efficacy against oral viral challenge in mice. Vaccine.

[51] Li H.Y., Han J.F., Qin C.F., Chen R. (2013). Virus-like particles for enterovirus 71 produced from Saccharomyces cerevisiae potently elicits protective immune responses in mice. Vaccine.

[52] Tsou Y.L., Lin Y.W., Shao H.Y., Yu S.L., Wu S.R., Lin H.Y., Liu C.C., Huang C., Chong P., Chow Y.H. (2015). Recombinant adeno-vaccine expressing enterovirus 71-like particles against hand, foot, and mouth disease. PLoS Negl. Trop. Dis..

[53] Yan Q., Wu L., Chen L., Qin Y., Pan Z., Chen M. (2016). Vesicular stomatitis virus-based vaccines expressing EV71 virus-like particles elicit strong immune responses and protect newborn mice from lethal challenges. Vaccine.

[54] Ventarola D., Bordone L., Silverberg N. (2015). Update on hand-foot-and-mouth disease. Clin. Dermatol..

[55] Zhang W., Dai W., Zhang C., Zhou Y., Xiong P., Wang S., Ye X., Liu Q., Zhou D., Huang Z. (2018). A virus-like particle-based tetravalent vaccine for hand, foot, and mouth disease elicits broad and balanced protective immunity. Emerg. Microbes Infect..

[56] Wu C.N., Lin Y.C., Fann C., Liao N.S., Shih S.R., Ho M.S. (2001). Protection against lethal enterovirus 71 infection in newborn mice by passive immunization with subunit VP1 vaccines and inactivated virus. Vaccine.

[57] Zhou S.L., Ying X.L., Han X., Sun X.X., Jin Q., Yang F. (2015). Characterization of the enterovirus 71 VP1 protein as a vaccine candidate. J. Med. Virol..

[58] Meng T., Kolpe A.B., Kiener T.K., Chow V.T., Kwang J. (2011). Display of VP1 on the surface of baculovirus and its immunogenicity against heterologous human enterovirus 71 strains in mice. PLoS ONE.

[59] Wang X., Dong K., Long M., Lin F., Gao Z., Wang L., Zhang Z., Chen X., Dai Y., Wang H. (2018). Induction of a high-titered antibody response using HIV gag-EV71 VP1-based virus-like particles with the capacity to protect newborn mice challenged with a lethal dose of enterovirus 71. Arch. Virol.

[60] Han X., Ying X.L., Zhou S.L., Han T., Huang H., Jin Q., Yang F., Sun Q.Y., Sun X.X. (2014). Characterization of the enterovirus 71 P1 polyprotein expressed in Pichia pastor as a candidate vaccine. Hum. Vaccin. Immunother.

[61] Fournillier A., Wychowski C., Boucreux D., Baumert T.F., Meunier J.C., Jacobs D., Muguet S., Depla E., Inchauspe G. (2001). Induction of hepatitis C virus E1 envelope protein-specific immune response can be enhanced by mutation of N-glycosylation sites. J. Virol..

[62] Chakrabarti B.K., Kong W.P., Wu B.Y., Yang Z.Y., Friborg J., Ling X., King S.R., Montefiori D.C., Nabel G.J. (2002). Modifications of the human immunodeficiency virus envelope glycoprotein enhance immunogenicity for genetic immunization. J. Virol..

[63] Foo D.G., Alonso S., Phoon M.C., Ramachandran N.P., Chow V.T., Poh C.L. (2007). Identification of neutralizing linear epitopes from the VP1 capsid protein of Enterovirus 71 using synthetic peptides. Virus Res..

[64] Foo D.G., Alonso S., Chow V.T., Poh C.L. (2007). Passive protection against lethal enterovirus 71 infection in newborn mice by neutralizing antibodies elicited by a synthetic peptide. Microbes. Infect..

[65] Liu J.N., Wang W., Duo J.Y., Hao Y., Ma C.M., Li W.B., Lin S.Z., Gao X.Z., Liu X.L., Xu Y.F. (2010). Combined peptides of human enterovirus 71 protect against virus infection in mice. Vaccine.

[66] Aw-Yong K.L., Sam I.C., Koh M.T., Chan Y.F. (2016). Immunodominant IgM and IgG Epitopes Recognized by Antibodies Induced in Enterovirus A71-Associated Hand, Foot and Mouth Disease Patients. PLoS ONE.

[67] Xu L., He D., Li Z., Zheng J., Yang L., Yu M., Yu H., Chen Y., Que Y., Shih J.W. (2014). Protection against lethal enterovirus 71 challenge in mice by a recombinant vaccine candidate containing a broadly cross-neutralizing epitope within the VP2 EF loop. Theranostics.

[68] Huo C., Yang J., Lei L., Qiao L., Xin J., Pan Z. (2017). Hepatitis B virus core particles containing multiple epitopes confer protection against enterovirus 71 and coxsackievirus A16 infection in mice. Vaccine.

[69] Jiang L., Fan R., Sun S., Fan P., Su W., Zhou Y., Gao F., Xu F., Kong W., Jiang C. (2015). A new EV71 VP3 epitope in norovirus P particle vector displays neutralizing activity and protection in vivo in mice. Vaccine.

[70] Mustafa S., Abd-Aziz N., Saw W.T., Liew S.Y., Yusoff K., Shafee N. (2020). Recombinant Enterovirus 71 Viral Protein 1 Fused to a Truncated Newcastle Disease Virus NP (NPt) Carrier Protein. Vaccines (Basel).

[71] Han J.F., Cao R.Y., Deng Y.Q., Tian X., Jiang T., Qin E.D., Qin C.F. (2011). Antibody dependent enhancement infection of enterovirus 71 in vitro and in vivo. Virol. J..

[72] Wang X., Peng W., Ren J., Hu Z., Xu J., Lou Z., Li X., Yin W., Shen X., Porta C. (2012). A sensor-adaptor mechanism for enterovirus uncoating from structures of EV71. Nat. Struct. Mol. Biol..

[73] Pardi N., Hogan M.J., Porter F.W., Weissman D. (2018). mRNA vaccines—A new era in vaccinology. Nat. Rev. Drug Discov..

[74] Anderson E.J., Rouphael N.G., Widge A.T., Jackson L.A., Roberts P.C., Makhene M., Chappell J.D., Denison M.R., Stevens L.J., Pruijssers A.J. (2020). Safety and Immunogenicity of SARS-CoV-2 mRNA-1273 Vaccine in Older Adults. N. Engl. J. Med..

[75] Widge A.T., Rouphael N.G., Jackson L.A., Anderson E.J., Roberts P.C., Makhene M., Chappell J.D., Denison M.R., Stevens L.J., Pruijssers A.J. (2020). Durability of Responses after SARS-CoV-2 mRNA-1273 Vaccination. N. Engl. J. Med..

